# The genus *Pareuryaptus* (Carabidae, Pterostichini) in China, with three new country records

**DOI:** 10.3897/BDJ.10.e84104

**Published:** 2022-05-30

**Authors:** Haoyuan Li, Yihang Li, Hongliang Shi, Hongbin Liang

**Affiliations:** 1 College of Life Science, Capital Normal University, Beijing, China College of Life Science, Capital Normal University Beijing China; 2 College of Agriculture, Purdue University, West Lafayette, United States of America College of Agriculture, Purdue University West Lafayette United States of America; 3 College of Forestry, Beijing Forestry University, Beijing, China College of Forestry, Beijing Forestry University Beijing China; 4 Institute of Zoology, Chinese Academy of Sciences, Beijing, China Institute of Zoology, Chinese Academy of Sciences Beijing China

**Keywords:** Trigonotomina, *Pareuryaptus*, key, new record, China

## Abstract

**Background:**

*Pareuryaptus* is a genus of Carabidae containing 18 species and one subspecies, distributed mainly in the Oriental Region. However, only one species and one subspecies were recorded from China before the present study.

**New information:**

Four species and one subspecies of *Pareuryaptus* are reported from China with three of them newly recorded: *Pareuryaptusadoxus* (Tschitschérine) from Yunnan; *Pareuryaptusexiguus* Dubault, Lassalle & Roux from Guangxi; and *Pareuryaptusluangphabangensis* Kirschenhofer from Yunnan. Moreover, the male of *Pareuryaptusexiguus* Dubault, Lassalle & Roux is firstly described and a key to all known Chinese species is provided.

## Introduction

*Pareuryaptus* is a genus under the subtribe Trigonotomina (Carabidae, Pterostichini). Dubault et al. (2008a) established the genus *Pareuryaptus* to accommodate some species previously placed in genus *Trigonotoma* Dejean and described several new species. A total of 18 species and one subspecies are known in the genus, mainly distributed in the Oriental Realm. However, only one species and one subspecies were recorded from China before the present study.

It is not surprising for us to find some new records of *Pareuryaptus* in China, because many species are distributed in China's southern neighbouring countries, such as Vietnam, Laos and Myanmar. We obtained a specimen from Xishuangbanna (South Yunnan) in the second half of 2020, which aroused our interest in this genus. After that, to make a more complete study, we collected more specimens and studied the collection of the Institute of Zoology, Chinese Academy of Sciences.

The primary purposes of this paper are to represent three new records of *Pareuryaptus* species from China, firstly to describe the male of *P.exiguus* that was originally described by a female holotype and to provide a key for all Chinese *Pareuryaptus* species.

## Materials and methods

The specimens examined in the present study are from these collections:

- **CHLC** Collection of Haoyuan Li, Beijing, China.
- **CJCC** Collection of Jiaheng Chen, Guangdong, China.
- **CYLC** Collection of Yihang Li, Beijing, China.
- **IZAS** Institute of Zoology, Chinese Academy of Sciences, Beijing, China.

In the citations of species, the following abbreviations of collections were mentioned, but there is no specimen examination from these collections:

- **CADA** Collection of A. Dostal, Vienna, Austria.
- **MNHN** Museum National d’Histoire Naturelle, Paris, France.
- **NMPC** Narodni Muzeum Prirodovedecke Muzeum, Prague, Czech Republic.
- **ZMAS** Zoological Institute, Russian Academy of Sciences, Saint Petersburg, Russia.

Habitus and aedeagus were captured by a Nikon D7200 camera with LAOWA 60 mm F2.8 2:1 Super Macro Lens. Maxillary palpi and pronota were captured by a Nikon D5500 camera with a Nikon SMZ18 stereomicroscope. Stains and dust on specimens were moderately cleared using Photoshop Elements 2022 Editor 20.0 after photographing.

## Taxon treatments

### Pareuryaptus

Dubault, Lassalle & Roux, 2008

F32101B3-C4D6-59BB-9CC0-EA518A63AE69

#### Type species

*Trigonotomacurtula* Chaudoir 1868

#### Diagnosis

Amongst the seven genera (*Trigonotoma* Dejean, *Lesticus* Dejean, *Euryaptus* Bates, *Nesites* Andrews, *Pareuryaptus* Dubault, Lassalle & Roux, *Leiolesticus* Roux, Lassalle & Dubault and *Trigonaptus* Fedorenko) of Trigonotomina, *Pareuryaptus* can be distinguished by the following character combinations: first antennomere (scape) longer than the length of the second and third antennomeres combined; antennal pedicel glabrous, without ventral seta; apex of labrum emarginate, with six apical setae, four grouped near the middle, the lateral setae evidently distant from the median four ones; parascutellar striae absent or very short; metacoxa unisetose; females with one seta on each side of sternite VII (Roux et al. 2016, Fedorenko 2020).

### Pareuryaptus adoxus

(Tschitschérine, 1900)

835149AF-1F4D-5112-972C-6742CDCC6FF1

Tschitschérine (1900): 162 (original: *Trigonotoma*; type locality: Saigon; holotype in ZMAS); Lorenz (2005): 300 (*Trigonotoma*, catalogue); Dubault et al. (2008a): 241; Dubault et al. (2008b): 199; Roux et al. (2016): 52.

#### Materials

- **Type status:** Other material. **Occurrence:** recordedBy: Chunpei Hong; individualCount: 1; sex: female; **Taxon:** scientificName: *Pareuryaptusadoxus* (Tschitschérine, 1900); **Location:** country: China; stateProvince: Yunnan; verbatimLocality: Xishuangbanna, Damenglong; verbatimElevation: 650 m; **Event:** year: 1958; month: 7; day: 12; **Record Level:** institutionCode: IZAS
- **Type status:** Other material. **Occurrence:** recordedBy: L. Z. Meng; individualCount: 1; sex: female; **Taxon:** scientificName: *Pareuryaptusadoxus* (Tschitschérine, 1900); **Location:** country: China; stateProvince: Yunnan; verbatimLocality: Jinghong, Nabanhe Natural Reserve, Mengsong Country, Danuoyou; verbatimElevation: 770 m; verbatimLatitude: 22.20699°N; verbatimLongitude: 100.63761°E; **Event:** samplingProtocol: pitfall trap; year: 2009; month: 5; day: 26; **Record Level:** institutionCode: IZAS

#### Diagnosis

Habitus: Fig. 1a. Maxillary palpus: Fig. 3a. Pronotum: Fig. 4a.

Body length = 12.2–12.6 mm. Dorsal surface largely black. Terminal maxillary palpimere cylindrical, of similar width as distal end of penultimate one, distinctly longer than penultimate one. Pronotum faintly blue, transversal round (pronotum width / pronotum length = 1.40–1.42), widest a little behind anterior third; pronotum densely and coarsely punctate between basal foveae; lateral margins hardly sinuate in front of posterior angles; posterior angles rounded and obtuse.

This species can be well distinguished from other Chinese species by the terminal maxillary palpimere cylindrical, not wider, but much longer than the penultimate one.

#### Remarks

Xishuangbanna, the collection locality of the two specimens we examined, is quite far from South Vietnam, the type locality of *P.adoxus*. However, we are confident in determining them as *P.adoxus* because all characteristics are nearly identical to the description and images of this species (Roux et al. 2016).

#### Distribution

Vietnam, Laos and a new record for China (Yunnan): Fig. 5.

### Pareuryaptus exiguus

Dubault, Lassalle & Roux, 2008

6C816D3D-932F-551C-80A4-325FA0B4C054

Dubault et al. (2008b): 201 (type locality: Hagiang; holotype in MNHN); Roux et al. (2016): 60.

#### Materials

- **Type status:** Other material. **Occurrence:** recordedBy: Feng Zegang; individualCount: 1; sex: male; **Taxon:** scientificName: *Pareuryaptusexiguus* Dubault, Lassalle & Roux, 2008; **Location:** country: China; stateProvince: Guangxi; verbatimLocality: Fangcheng District, Nasuo Town, Naqin Village, 248 platform; verbatimElevation: 852 m; verbatimLatitude: N21.7669; verbatimLongitude: E108.0598; **Event:** samplingProtocol: soil sieve; year: 2020; month: 12; day: 10; **Record Level:** institutionCode: IZAS
- **Type status:** Other material. **Occurrence:** recordedBy: Subai Liao; individualCount: 1; sex: female; **Taxon:** scientificName: *Pareuryaptusexiguus* Dubault, Lassalle & Roux, 2008; **Location:** country: China; stateProvince: Guangxi; verbatimLocality: Daqing Mt.; verbatimElevation: 700 m; **Event:** year: 1983; month: 5; day: 5; **Record Level:** institutionCode: IZAS

#### Diagnosis

Habitus: Fig. 1b. Male genitalia: Fig. 2a, b. Maxillary palpus: Fig. 3b. Pronotum: Fig. 4b.

Body length = 12.6–13.7 mm. Dorsal surface dark brown to black. Terminal maxillary palpimere cylindrical, as long as penultimate one. Pronotum black, rounded, narrower than that of the previous species (pronotum width / pronotum length = 1.24–1.29), widest near middle; pronotum completely smooth between basal foveae; lateral margins hardly sinuate in front of posterior angles; posterior angles rounded and obtuse.

This species can be well distinguished from other Chinese species by the narrower pronotum and lateral margins evenly curved, widest near middle.

#### Supplementary descriptions.

Male genitalia: in lateral view, median lobe of aedeagus with ventral margin very weakly curved near middle, apical lamella nearly straight; in dorsal view, median lobe of aedeagus with both lateral margins sinuate, widest near basal third, gradually constricted near apical third, apex rounded with apical lamella very short, apical orifice opened left-dorsally.

#### Remarks

This species was originally described from a single female. We herein provide the first record of the male of this species and provide description and illustration for the male genitalia.

#### Distribution

Vietnam and a new record for China (Guangxi): Fig. 5.

### Pareuryaptus luangphabangensis

Kirschenhofer, 2011

7B06BA97-3D6B-52AF-BC16-42D53CE72681

Kirschenhofer (2011): 33 (type locality: Luang Phabang; holotype in CADA); Roux et al. (2016): 78.

#### Materials

- **Type status:** Other material. **Occurrence:** recordedBy: Zheng Guo; individualCount: 1; sex: male; **Taxon:** scientificName: *Pareuryaptusluangphabangensis* Kirschenhofer, 2011; **Location:** country: China; stateProvince: Yunnan; verbatimLocality: Jinghong Menglun xishuangbanna P G 2 – 19; verbatimDepth: 558 m; verbatimLatitude: N: 21°55.035'; verbatimLongitude: E: 101°16.500'; **Event:** year: 2007; month: 5; day: 10; **Record Level:** institutionCode: IZAS
- **Type status:** Other material. **Occurrence:** recordedBy: Yi Li; individualCount: 1; sex: male; **Taxon:** scientificName: *Pareuryaptusluangphabangensis* Kirschenhofer, 2011; **Location:** country: China; stateProvince: Yunnan; verbatimLocality: Xishuangbanna Dai Autonomous Prefecture, Jinghong City, Jino Mt.; verbatimLatitude: 22.0373N; verbatimLongitude: 101.0044E; **Event:** year: 2020; month: 5; day: 18-2; **Record Level:** collectionCode: CHLC
- **Type status:** Other material. **Occurrence:** recordedBy: Hui Ce; individualCount: 2; sex: 1 male, 1 female; **Taxon:** scientificName: *Pareuryaptusluangphabangensis* Kirschenhofer, 2011; **Location:** country: China; stateProvince: Yunnan; verbatimLocality: Xishuangbanna Dai Autonomous Prefecture, Jinghong City, Gasa Town, Nanpaxiaozhai; verbatimElevation: 1000 m; **Event:** year: 2021; month: 3-5; **Record Level:** collectionCode: CHLC

#### Diagnosis

Habitus: Fig. 1c, d. Male genitalia: Fig. 2c, d. Maxillary palpus: Fig. 3c, d. Pronotum: Fig. 4c.

Body length = 12.8–14.6 mm. Dorsal surface black. Terminal maxillary palpimere cylindrical, as long as penultimate one. Pronotum black, widely cordate (pronotum width / pronotum length = 1.37–1.44), widest a little behind anterior third; pronotum completely smooth between basal foveae; lateral margins strongly sinuate in front of posterior angles; posterior angles rectangular with sharp apices.

This species can be well distinguished from other Chinese species by its characteristic pronotum that is strongly cordate with evident sinuation in front of posterior angles, which are sharp.

#### Remarks

This species is somewhat similar to *P.aethiops* distributed in Myanmar. Based on the descriptions in literature (Roux et al. 2016), these two species are mainly different in the shape of pronotum: lateral margins evidently sinuate before posterior angles in *P.luangphabangensis*, but hardly sinuate in *P.aethiops*; apex of posterior angles sharp in *P.luangphabangensis*, but nearly rounded in *P.aethiops*.

#### Distribution

Laos and a new record for China (Yunnan): Fig. 5.

### Pareuryaptus chalceolus chalceolus

(Bates, 1873)

B12CFBC9-B933-590B-A39B-94F53B6FBEB0

Bates (1873): 328 (original: *Trigonotoma*; type locality: Hong Kong; lectotype in MNHN); Bates (1889): 16 (*Trigonotoma*, Saigon); Lorenz (2005): 300 (*Trigonotoma*, catalogue); Dubault et al. (2007): 216 (*Trigonotoma*); Dubault et al. (2008a): 241; Dubault et al. (2008b): 207; Roux et al. (2016): 84; Löbl and Löbl (2017): 688 (catalogue).
Synonym: *Trigonotomaannamensis* Jedlička (1962): 313 (type locality: Cuatung, holotype in NMPC); Dubault et al. (2008b): 208 (synonymised to *P.chalceolus*).

#### Materials

- **Type status:** Other material. **Occurrence:** recordedBy: A de cooman; individualCount: 1; sex: male; **Taxon:** scientificName: *Pareuryaptuschalceoluschalceolus* (Bates, 1873); **Location:** country: Vietnam; verbatimLocality: TONKIN Hoa Binh; **Record Level:** institutionCode: IZAS
- **Type status:** Other material. **Occurrence:** recordedBy: Subai Liao; individualCount: 1; sex: male; **Taxon:** scientificName: *Pareuryaptuschalceoluschalceolus* (Bates, 1873); **Location:** country: China; stateProvince: Guangxi; verbatimLocality: Daqing Mt.; verbatimElevation: 700 m; **Event:** year: 1983; month: 5; day: 5; **Record Level:** institutionCode: IZAS
- **Type status:** Other material. **Occurrence:** recordedBy: Weifeng Yan; individualCount: 1; sex: male; **Taxon:** scientificName: *Pareuryaptuschalceoluschalceolus* (Bates, 1873); **Location:** country: China; stateProvince: Guangxi; verbatimLocality: Jingxi County, near Longbang Town Hotel; verbatimElevation: 700 m; verbatimLatitude: 22.879343N; verbatimLongitude: 106.330014E; **Event:** samplingProtocol: collect at night; year: 2018; month: 4; day: 29; **Record Level:** institutionCode: IZAS
- **Type status:** Other material. **Occurrence:** recordedBy: native collector; individualCount: 5; sex: 2 males, 3 females; **Taxon:** scientificName: *Pareuryaptuschalceoluschalceolus* (Bates, 1873); **Location:** country: China; stateProvince: Guangdong; verbatimLocality: Guangzhou City, Xinhui District, Guanyin Mt.; **Event:** year: 2021; month: 3; **Record Level:** collectionCode: CHLC
- **Type status:** Other material. **Occurrence:** recordedBy: native collector; individualCount: 1; sex: female; **Taxon:** scientificName: *Pareuryaptuschalceoluschalceolus* (Bates, 1873); **Location:** country: China; stateProvince: Fujian; verbatimLocality: Putian City, Hanjiang District; **Event:** year: 2020; month: 3; day: 10; **Record Level:** collectionCode: CJCC
- **Type status:** Other material. **Occurrence:** recordedBy: Jinse Song; individualCount: 2; sex: females; **Taxon:** scientificName: *Pareuryaptuschalceoluschalceolus* (Bates, 1873); **Location:** country: China; stateProvince: Hunan; verbatimLocality: Chenzhou City, Yizhang County, Yuxi Town; **Event:** year: 2021; month: 5; day: 1; **Record Level:** collectionCode: CYLC

#### Diagnosis

Habitus: Fig. 1e. Male genitalia: Fig. 2e, f. Maxillary palpus: Fig. 3e. Pronotum: Fig. 4e, f.

Body length = 11.9-14.5 mm. Dorsal surface black. Terminal maxillary palpimere elongated and ovate, much longer and wider than penultimate one. Pronotum black, widely rounded (pronotum width / pronotum length = 1.33–1.43), widest a little behind anterior third; pronotum densely punctate between basal foveae; lateral margins hardly sinuate in front of posterior angles; posterior angles obtuse.

This species can be well distinguished from other Chinese species by the terminal maxillary palpimere long-ovate, much longer and wider than the penultimate one.

#### Distribution

Vietnam and China (Hong Kong, Hainan, Guangxi, Guangdong, Fujian, Hunan): Fig. 5.

### Pareuryaptus chalceolus formosanus

(Jedlička, 1962)

0AB47281-4FD8-55A5-B0C3-495566718CE9

Jedlička (1962): 314 (original: *Trigonotomaformosanus*; type locality: Takao; holotype in NMPC); Lorenz (2005): 300 (*Trigonotomaformosanus*, catalogue); Dubault et al. (2008a): 241 (*Pareuryaptusformosanus*); Dubault et al. (2008b): 208 (as subspecies of *P.chalceolus*); Dubault et al. (2008c): 211 (*Pareuryaptusformosanus*); Roux et al. (2016): 84; Löbl and Löbl (2017): 688 (catalogue).

#### Materials

- **Type status:** Other material. **Occurrence:** recordedBy: Changchin Chen; individualCount: 1; sex: female; **Taxon:** scientificName: *Pareuryaptuschalceolusformosanus* (Jedlička, 1962); **Location:** country: China; stateProvince: Taiwan; verbatimLocality: Pingtung County, Jialeshui; **Event:** year: 2008; month: 6; day: 8; **Record Level:** institutionCode: IZAS

#### Diagnosis

Habitus: Fig. 1f. Maxillary palpus: Fig. 3f. Pronotum: Fig. 4d.

Body length = 11.7 mm. Dorsal surface black. Similar to the nominal-typical subspecies, but different in the pronotum being only sparsely punctate between basal foveae.

#### Distribution

China (Taiwan): Fig. 5.

## Identification Keys

### Key to Chinese species and subspecies of *Pareuryaptus*

**Table:** 

1	Pronotum more or less punctate between basal foveae; terminal maxillary palpimere much longer than penultimate one.	2
–	Pronotum completely smooth between basal foveae; terminal maxillary palpimere as long as penultimate one.	4
2	Terminal maxillary palpimere long-ovate, distinctly wider than distal end of penultimate one.	3
–	Terminal maxillary palpimere cylindrical, not wider than distal end of penultimate one.	*P.adoxus* (Tschitschérine, 1900)
3	Pronotum densely punctate between basal foveae, distributed in Mainland China.	*P.chalceoluschalceolus* (Bates, 1873)
–	Pronotum sparsely punctate between basal foveae, distributed in Taiwan, China.	*P.chalceolusformosanus* (Jedlička, 1962)
4	Pronotum lateral margins nearly straight before posterior angles, apex of posterior angles obtuse.	*P.exiguus* Dubault, Lassalle & Roux, 2008
–	Pronotum lateral margins strongly sinuate before posterior angles, apex of posterior angles sharp.	*P.luangphabangensis* Kirschenhofer, 2011

## Supplementary Material

XML Treatment for Pareuryaptus

XML Treatment for Pareuryaptus adoxus

XML Treatment for Pareuryaptus exiguus

XML Treatment for Pareuryaptus luangphabangensis

XML Treatment for Pareuryaptus chalceolus chalceolus

XML Treatment for Pareuryaptus chalceolus formosanus

## Figures and Tables

**Figure 1a.**
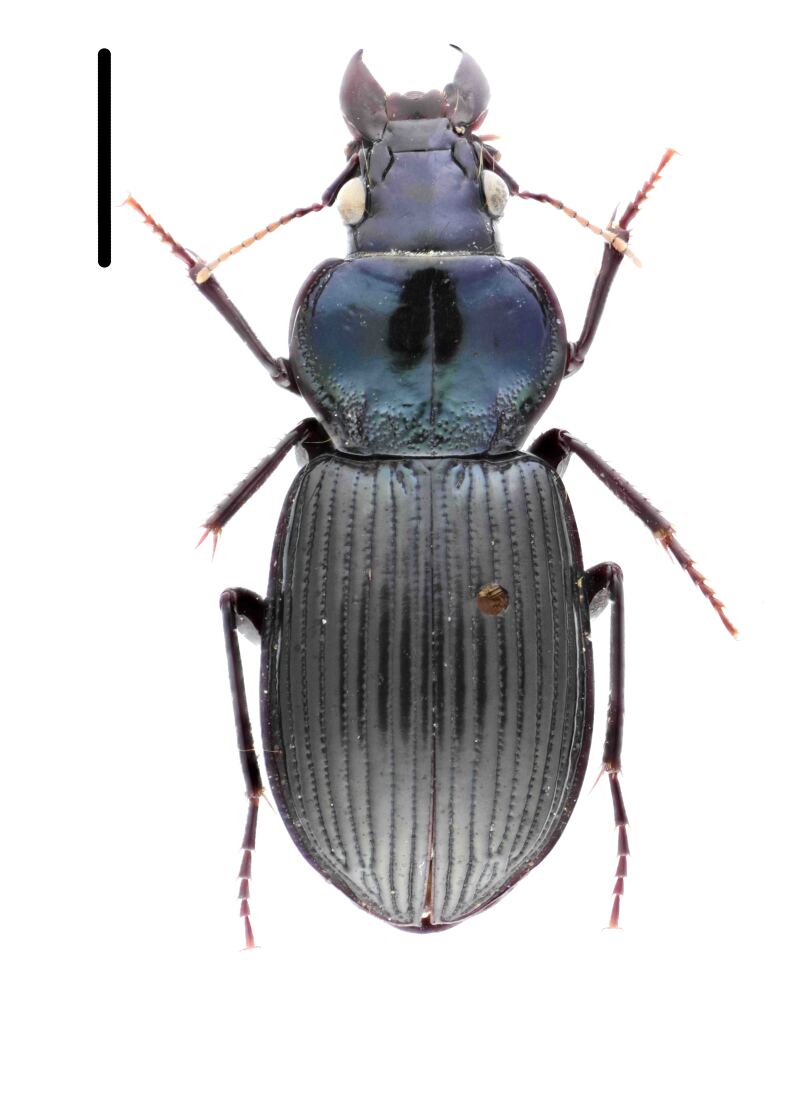


**Figure 1b.**
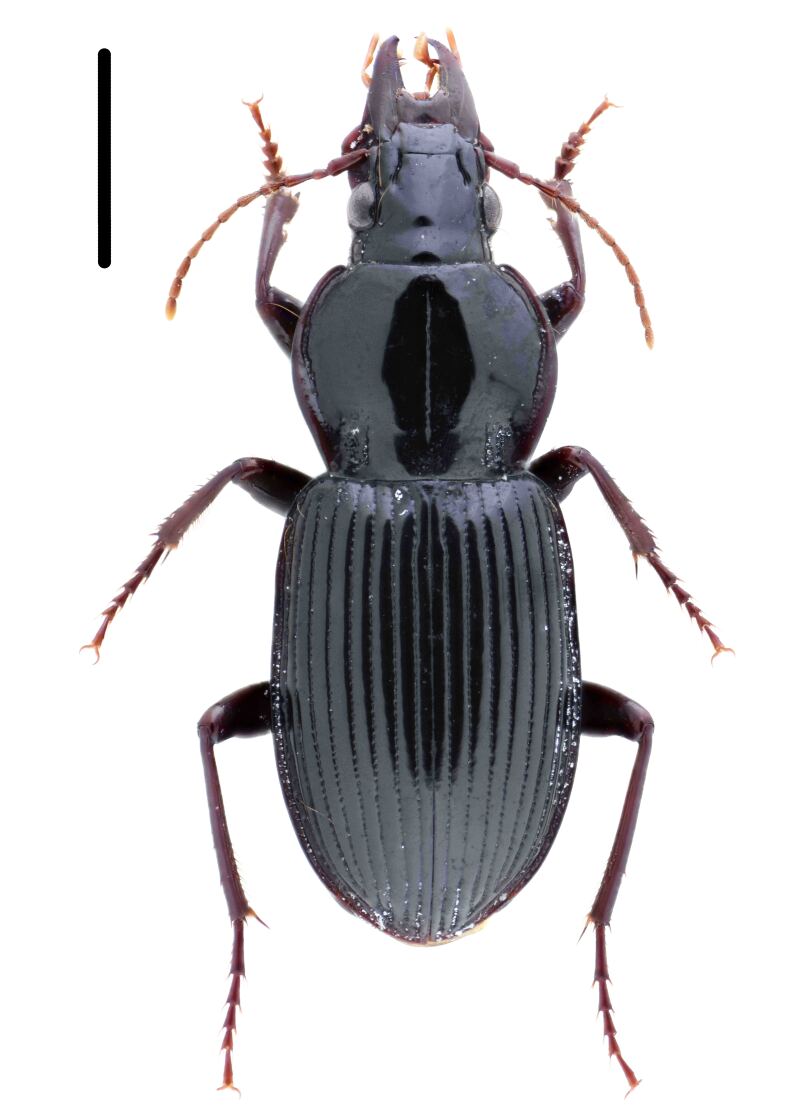


**Figure 1c.**
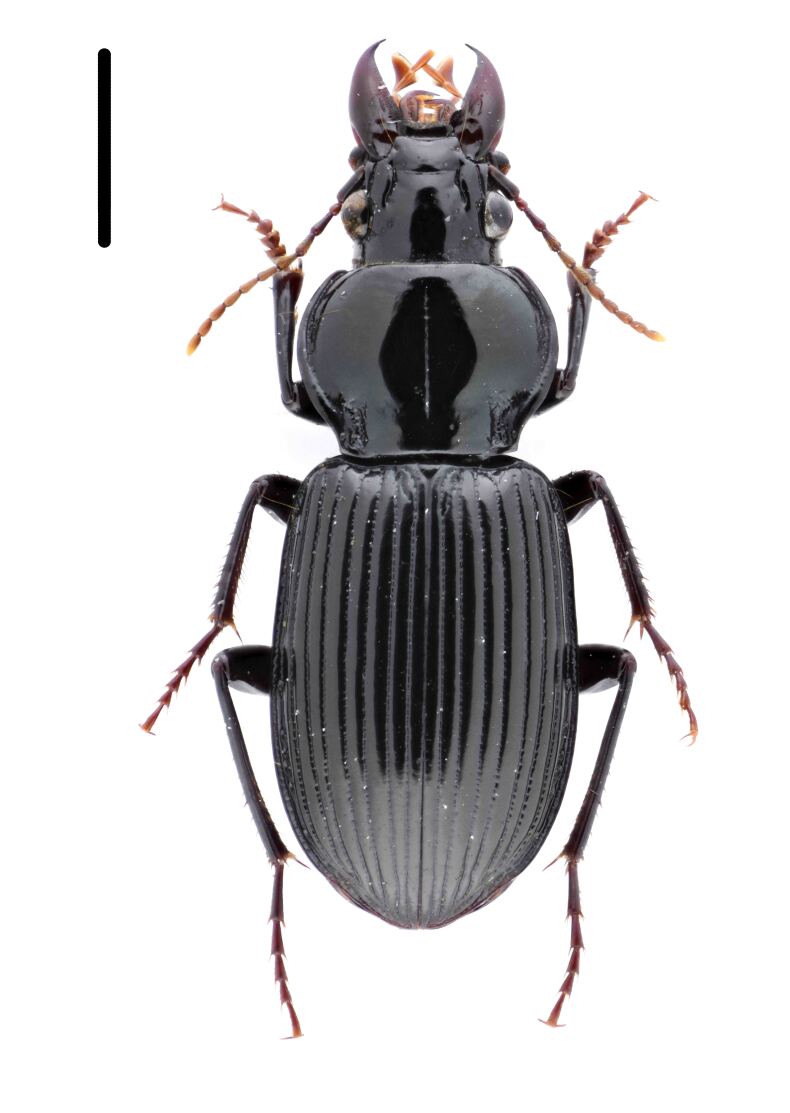


**Figure 1d.**
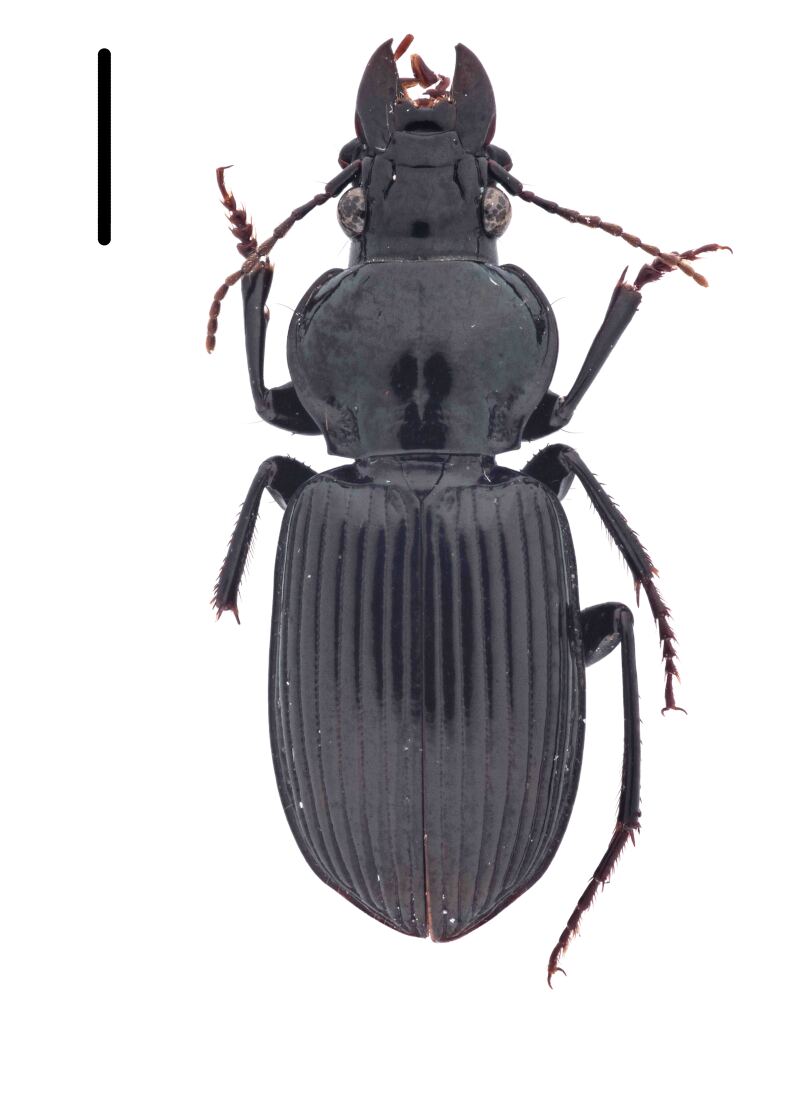


**Figure 1e.**
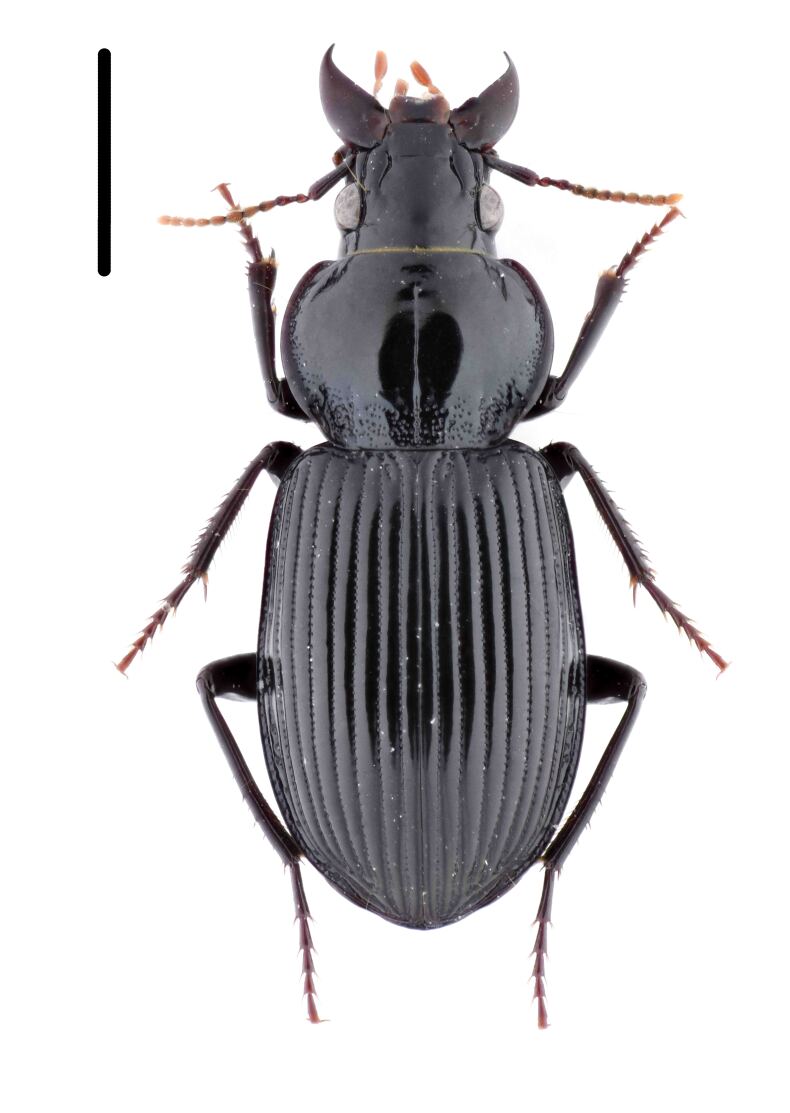


**Figure 1f.**
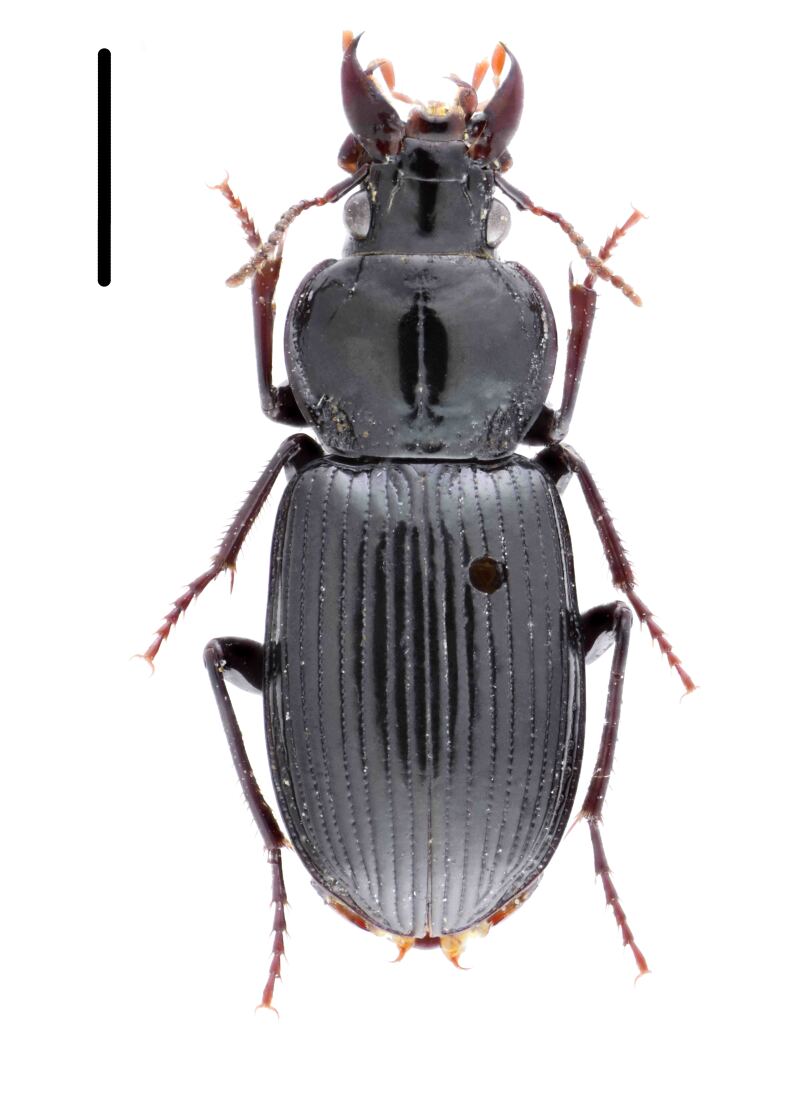


**Figure 2a.**
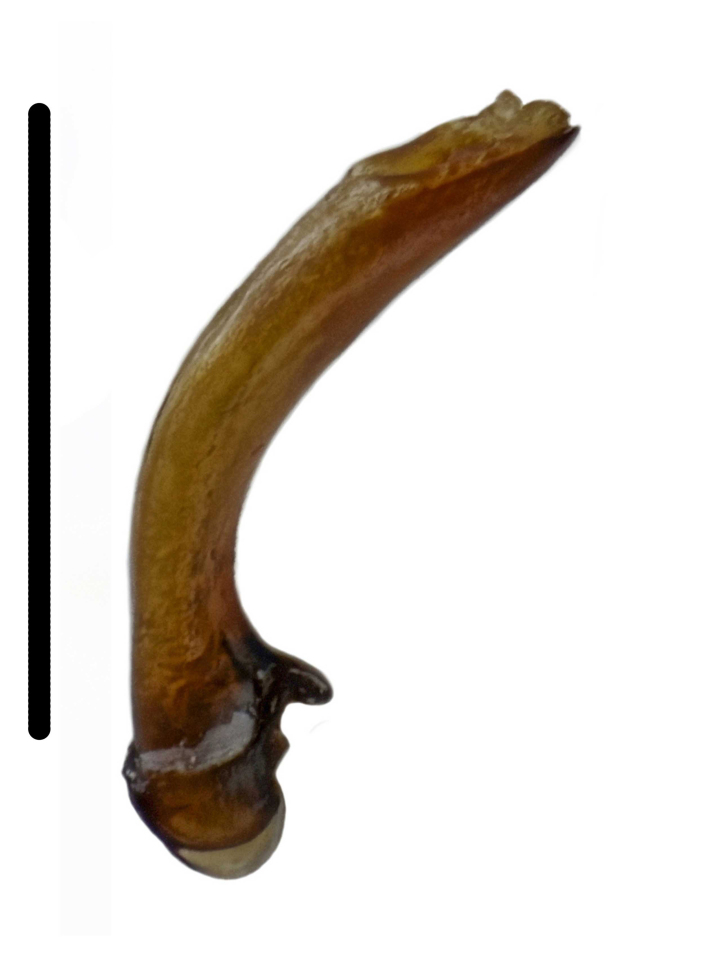


**Figure 2b.**
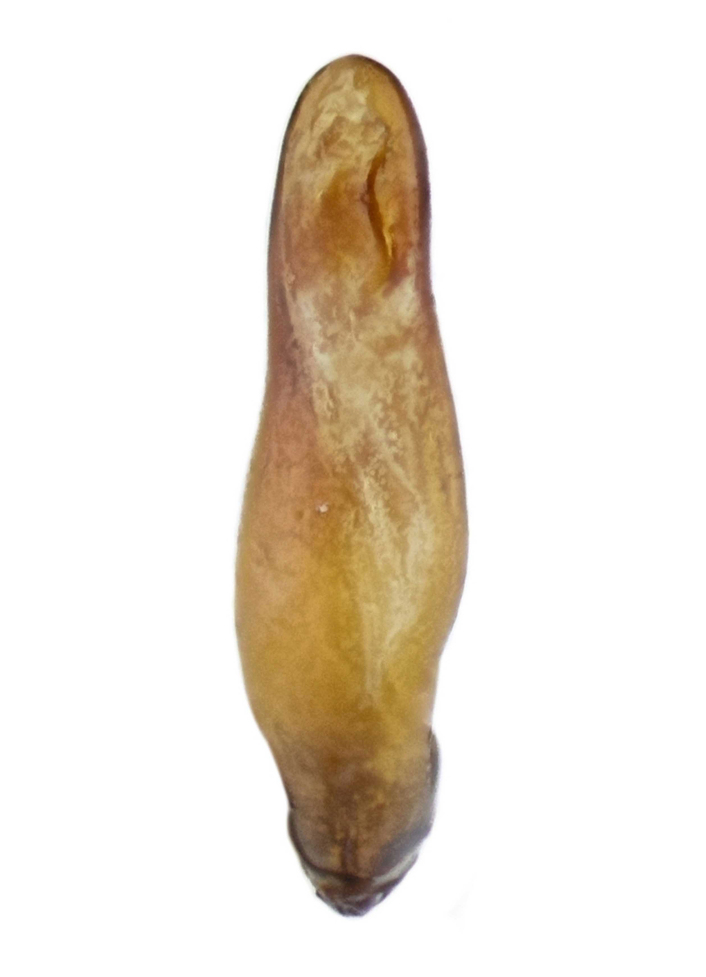


**Figure 2c.**
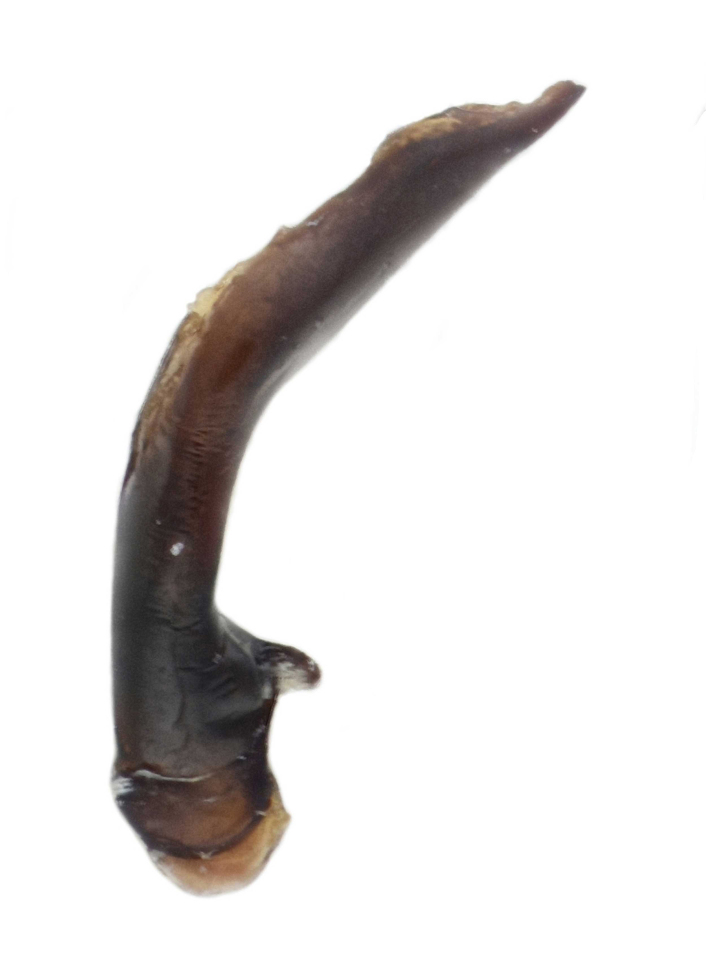


**Figure 2d.**
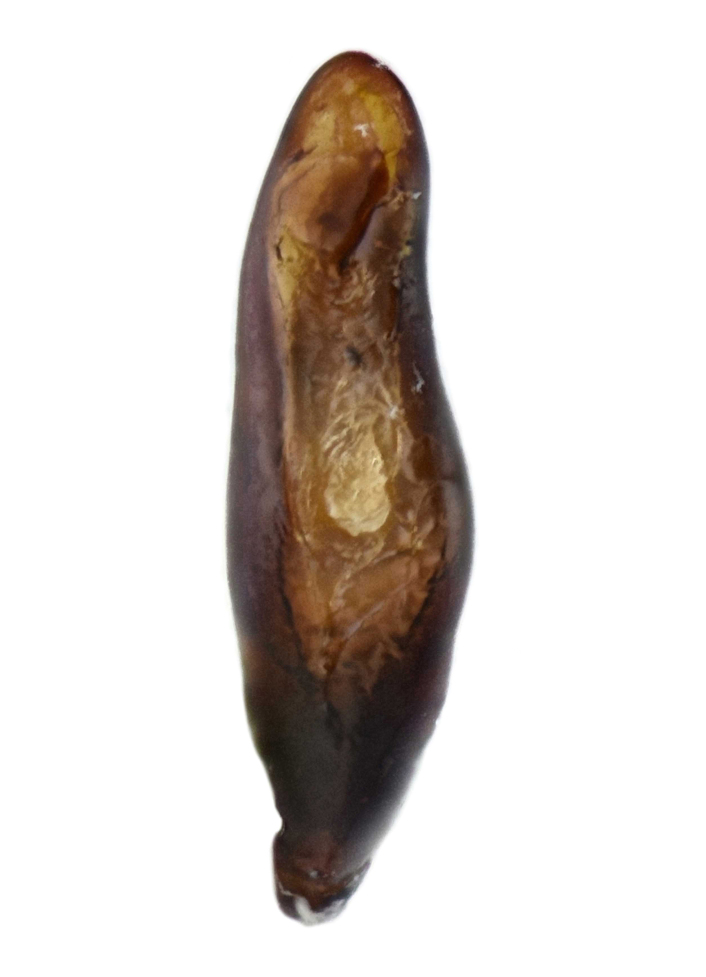


**Figure 2e.**
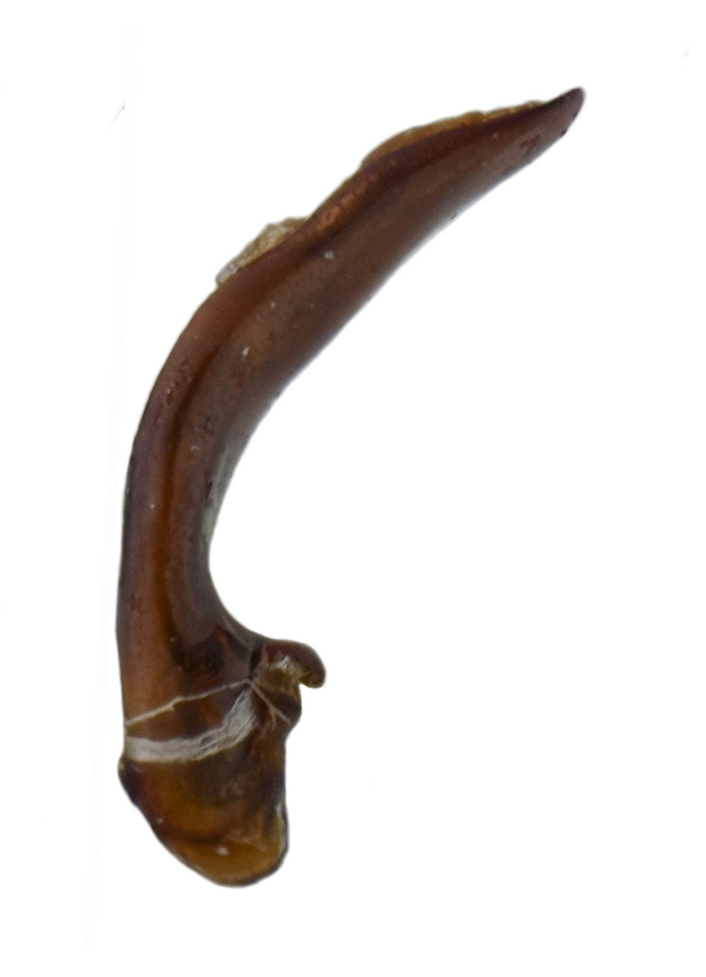


**Figure 2f.**
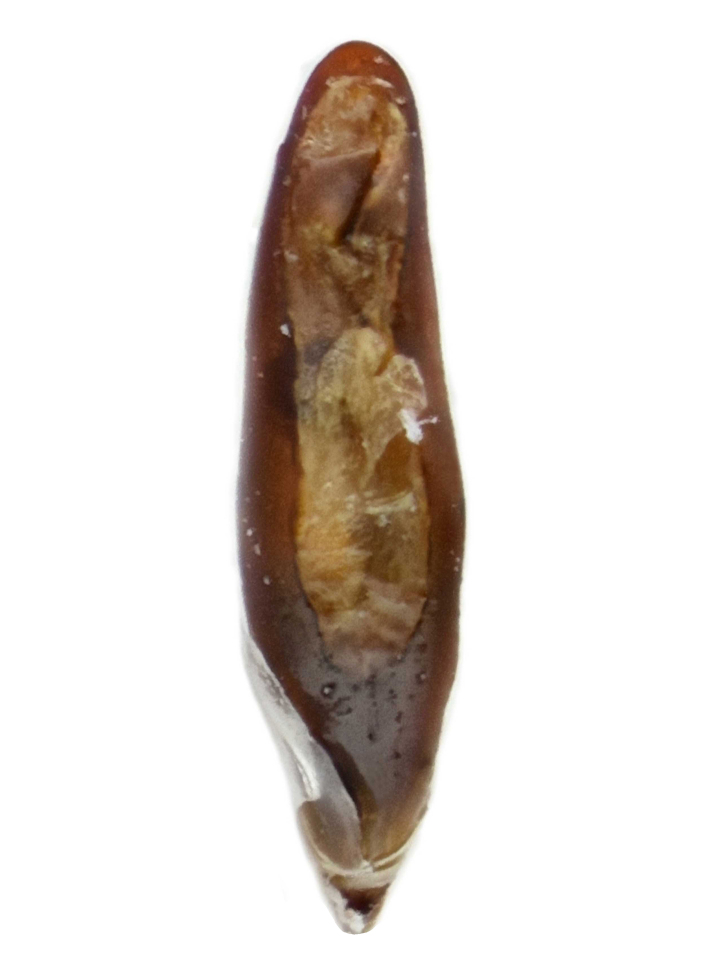


**Figure 3a.**
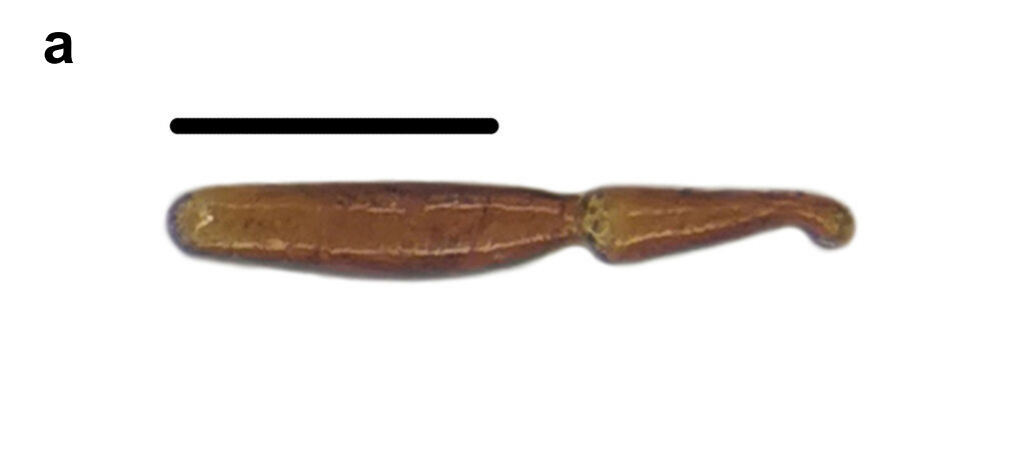


**Figure 3b.**
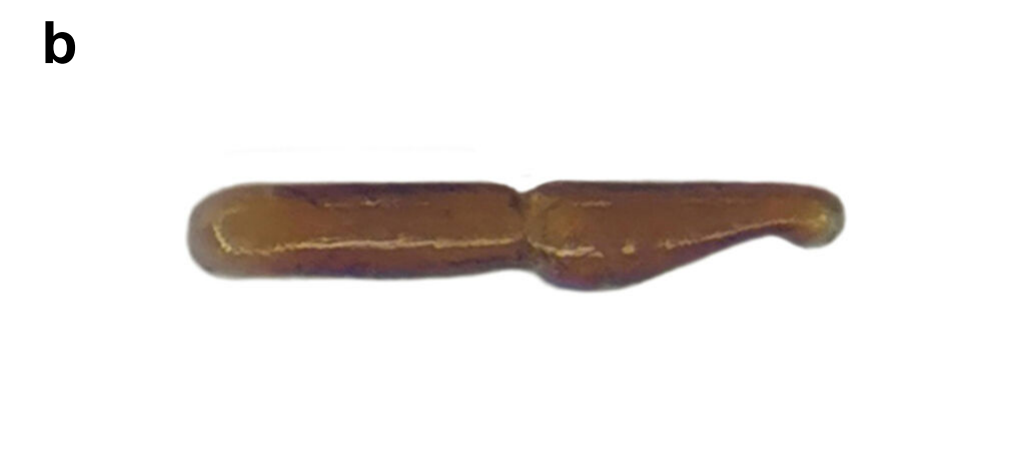


**Figure 3c.**
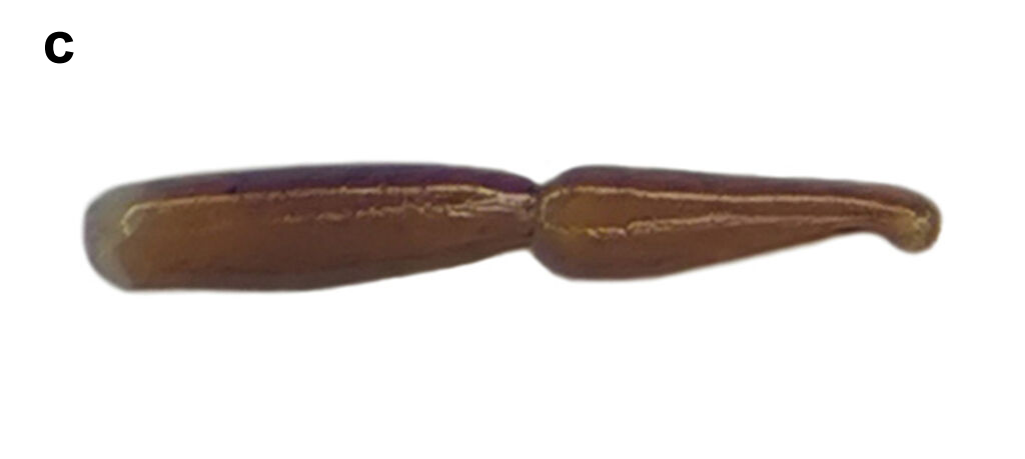


**Figure 3d.**
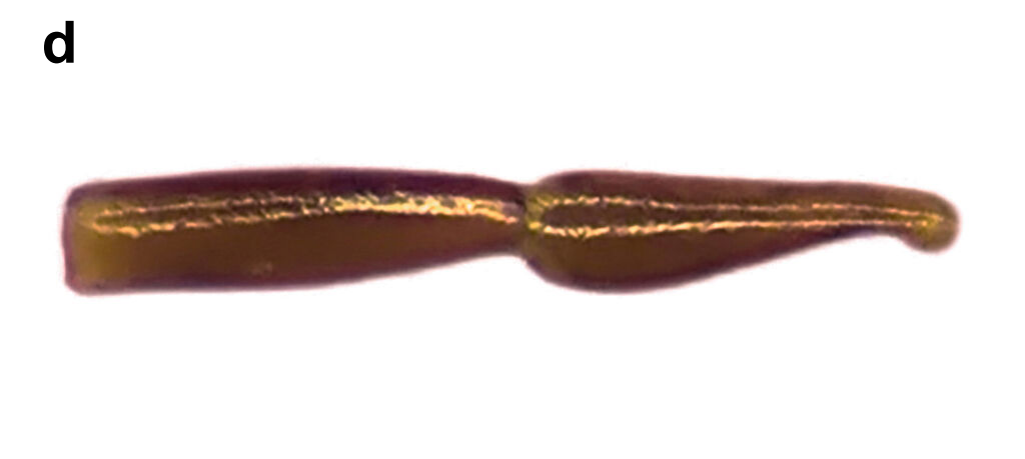


**Figure 3e.**
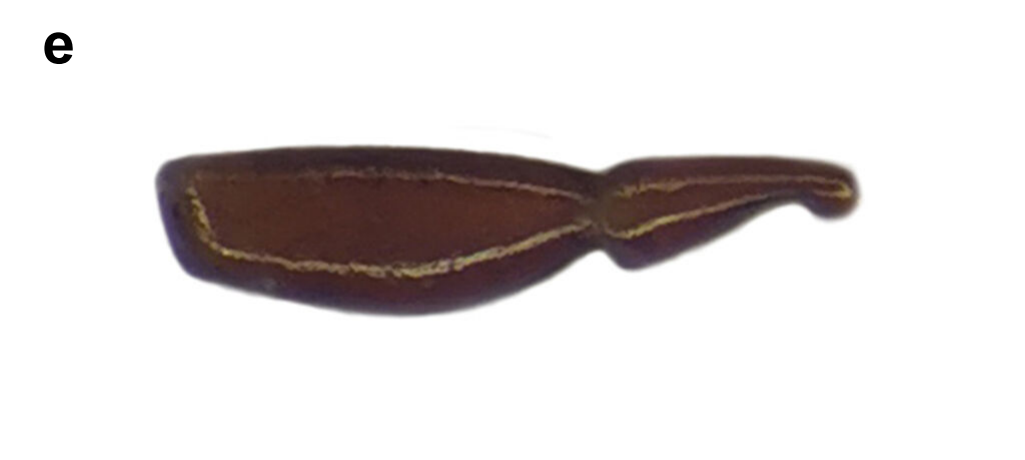


**Figure 3f.**
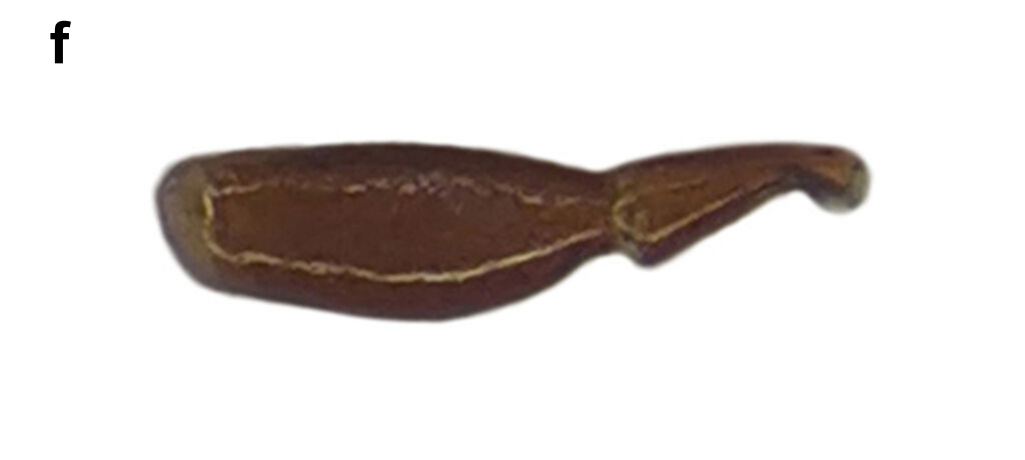


**Figure 4a.**
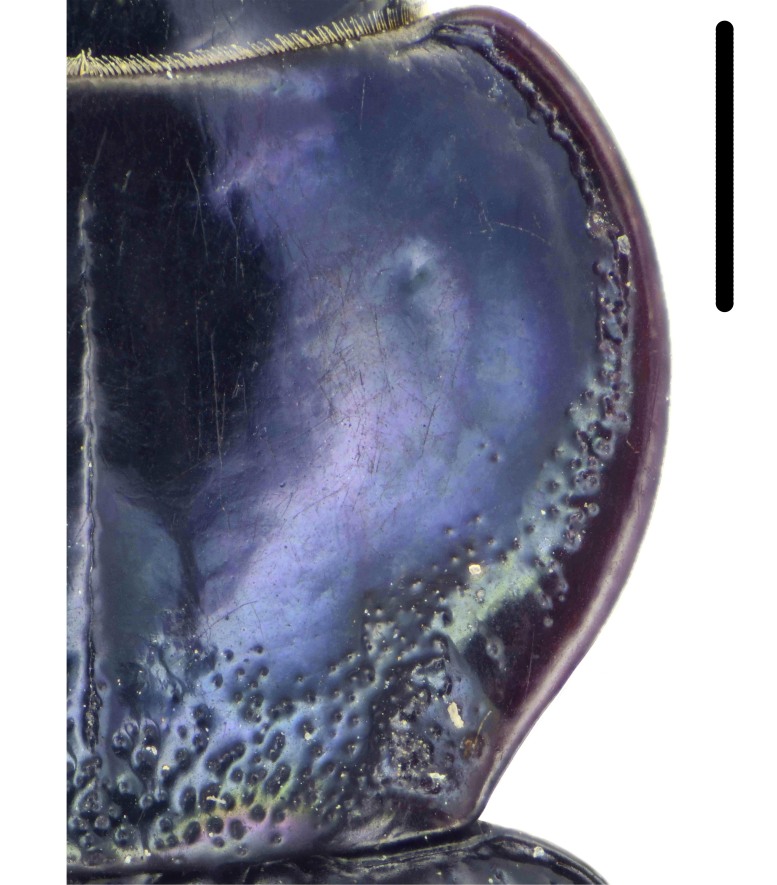


**Figure 4b.**
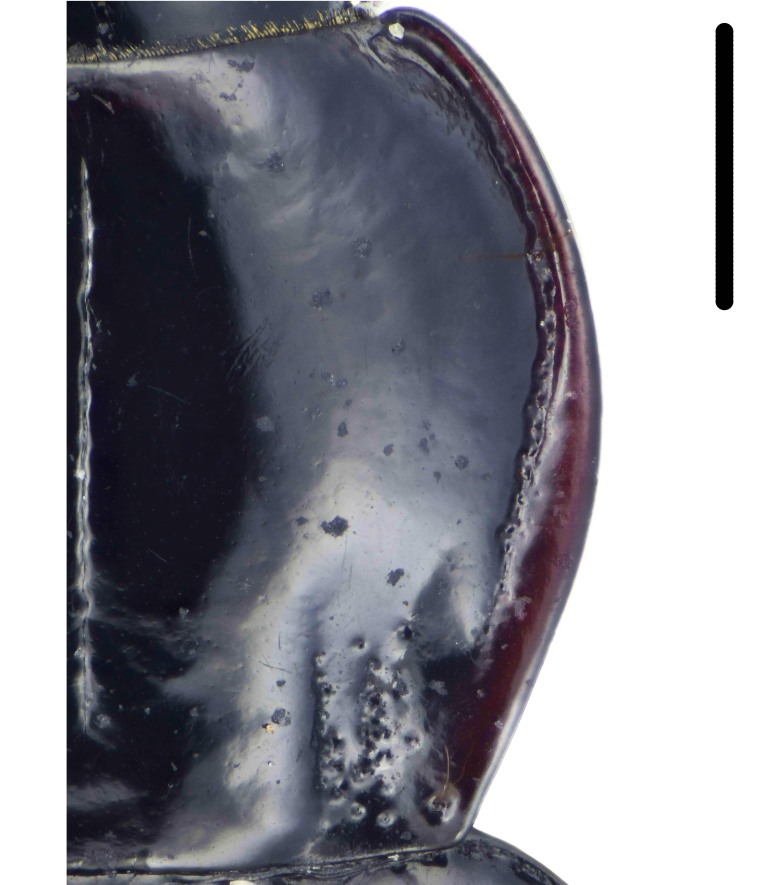


**Figure 4c.**
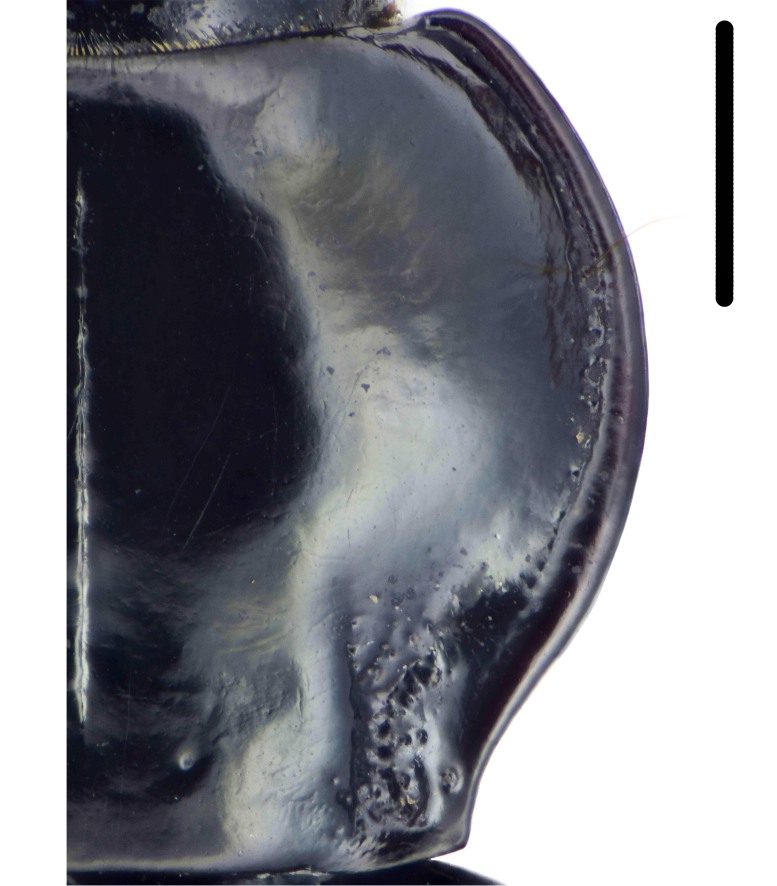


**Figure 4d.**
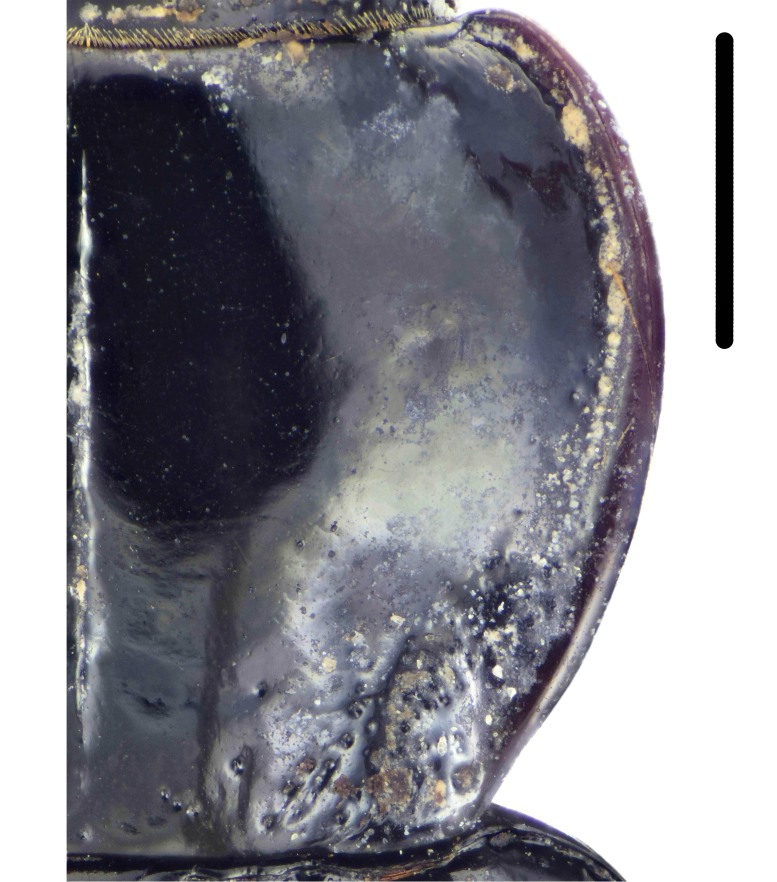


**Figure 4e.**
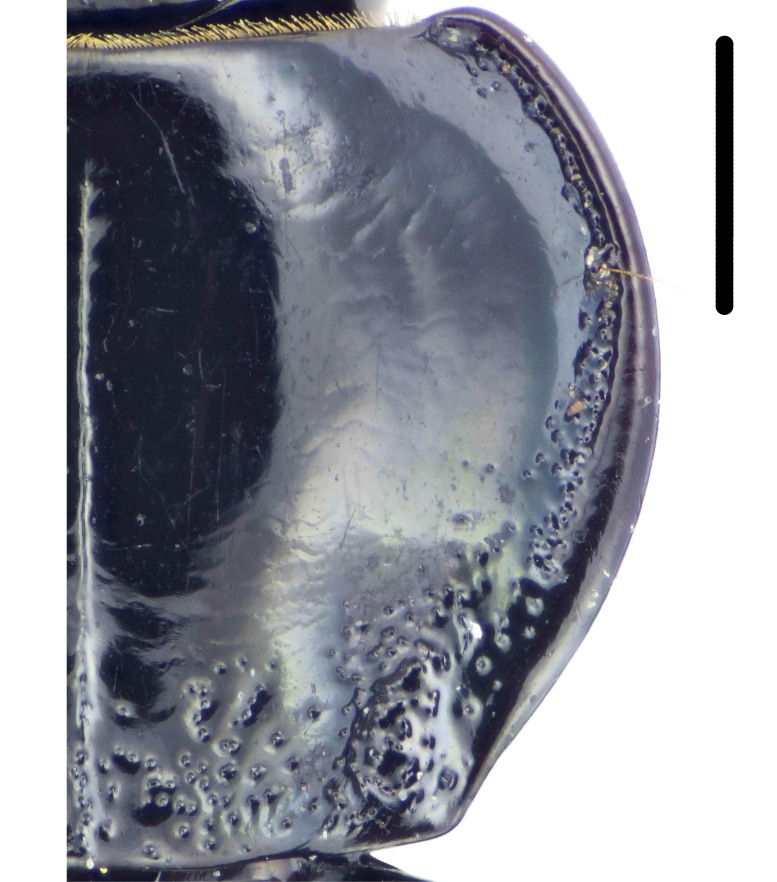


**Figure 4f.**
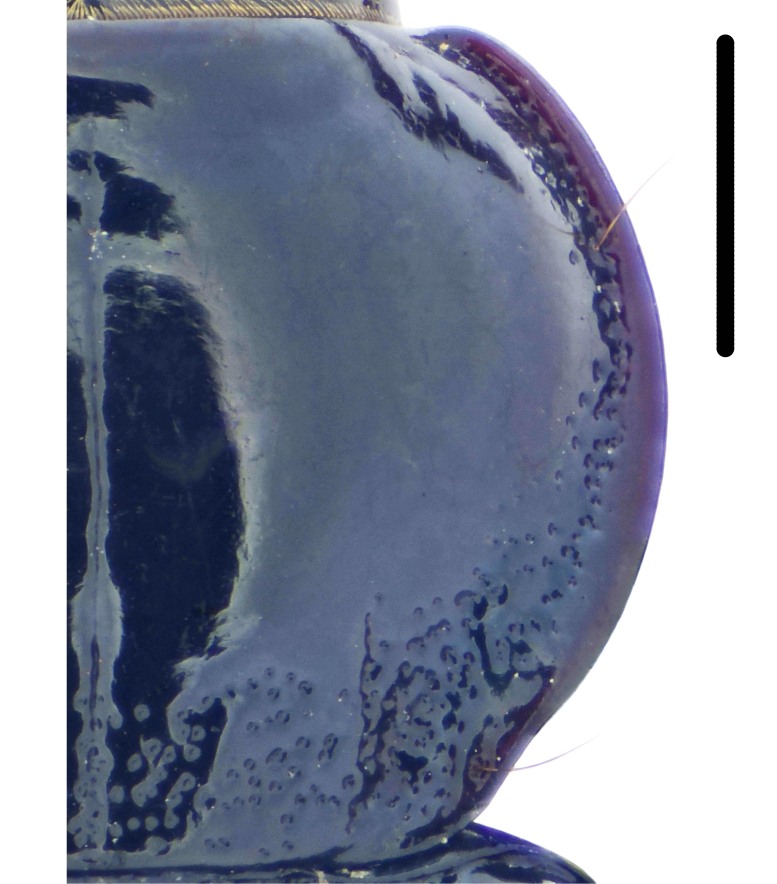


**Figure 5.**
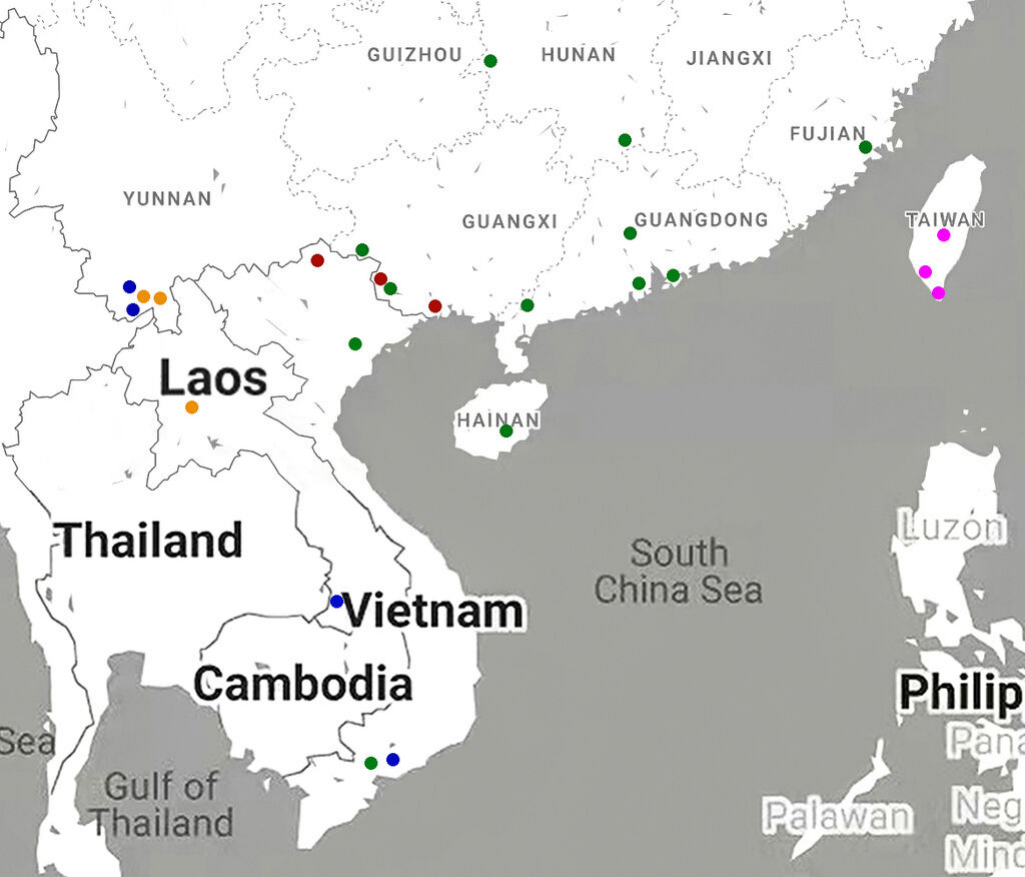
Distribution map for *Pareuryaptus* spp. known from China: *P.adoxus* (Tschitschérine) (blue); *P.exiguus* Dubault, Lassalle & Roux (red); *P.luangphabangensis* Kirschenhofer (orange); *P.chalceoluschalceolus* (Bates) (green); *P.chalceolusformosanus* (Jedlička) (magenta).
